# The relationship between knowledge of leadership and knowledge management practices in the food industry in Kurdistan province, Iran

**DOI:** 10.1016/j.dib.2017.09.031

**Published:** 2017-09-22

**Authors:** Seyyed Mohammad Moosavi Jad, Sahar Geravandi, Mohammad Javad Mohammadi, Rashin Alizadeh, Mohammad Sarvarian, Babak Rastegarimehr, Abolhasan Afkar, Ahmad Reza Yari, Mahboobeh Momtazan, Aliasghar Valipour, Mohammad Mahboubi, Azimeh Karimyan, Alireza Mazraehkar, Ali Soleimani Nejad, Hafez Mohammadi

**Affiliations:** a Faculty of Humanities and Social Sciences, Kurdistan University, Sanandaj, Iran; b Razi Teaching Hospital, Clinical Research Development Center, Razi Hospital, Ahvaz Jundishapur University of Medical Sciences, Ahvaz, Iran; c Abadan school of Medical Sciences, Abadan, Iran; d Department of Health Education and Promotion, School of Health, Tehran University of Medical Sciences, Tehran, Iran; e Social Determinants of Health Research Center, Guilan University of Medical Sciences, Rasht, Iran; f Research Center for Environmental Pollutants, Qom University of Medical Sciences, Qom, Iran; g Faculty of Humanities and Social Sciences, Executive management-Strategic, Sanandaj Branch, Islamic Azad University, Sanandaj, Iran

**Keywords:** Kurdistan FI, Kurdistan food industry, SCC, Spearman correlation coefficient, KM, knowledge management, OP, organizational performance, ME, middle east, Knowledge-oriented leadership, Product innovation performance, Knowledge management practices, Iran

## Abstract

The aim of this study was to identify the relationship between the knowledge of leadership and knowledge management practices. This research strategy, in terms of quantity, procedure and obtain information, is descriptive and correlational. Statistical population, consist of all employees of a food industry in Kurdistan province of Iran, who were engaged in 2016 and their total number is about 1800 people. 316 employees in the Kurdistan food industry (Kurdistan FI) were selected, using Cochran formula. Non-random method and valid questions (standard) for measurement of the data are used. Reliability and validity were confirmed. Statistical analysis of the data was carried out, using SPSS 16. The statistical analysis of collected data showed the relationship between knowledge-oriented of leadership and knowledge management activities as mediator variables. The results of the data and test hypotheses suggest that knowledge management activities play an important role in the functioning of product innovation and the results showed that the activities of Knowledge Management (knowledge transfer, storage knowledge, application of knowledge, creation of knowledge) on performance of product innovation.

**Specifications Table**

**Table:** 

Subject area	*Business, Management and Accounting*
More specific subject area	*knowledge leadership and knowledge management practices*
Type of data	*Table, figure*
How data was acquired	*researcher-made questionnaire analysis*
Data format	*Raw, analyzed, Descriptive and statistical data*
Experimental factors	– *Sample consisted of employees in the Kurdistan food industry.* – *After Inviting the employer, the researcher-made questionnaire including demographic data as well as the knowledge of leadership and knowledge management practices questionnaires were completed.* – *In this paper, the effects of knowledge management practices on improved knowledge of leadership have been studied.*
Experimental features	*Knowledge* of *leadership is one of the factors endangering the* efficiency.
Data source location	*Kurdistan, Iran*
Data accessibility	*Data is included in this article.*

**Value of the data**

• These data describe demographic data in employer food industry and affecting knowledge management activity on increase knowledge of leadership in order to control the organizational management.
• The results showed that the use of methods of knowledge management practices can be very helpful for organization leadership.
• The results of this study can be used to improved knowledge management practices and develop an employee satisfaction in the Kurdistan food industry.
• Results have also important role about knowledge leadership in order to improve the efficiency of work.

## 1 Data

Table 1 represents demographic characteristics of employees of the Kurdistan food industry of Iran during 2016 that used for description of experiments. The results showed that the highest educational qualification was in bachelor category (229(72.5%)). Also, based on result, the maximum of participants were men with 86.4%. Table 2 shows data for the relationship between knowledge of leadership and knowledge management practices in the Kurdistan food industry. According to the result, there was a significant relationship between the knowledge of leadership and knowledge management practices (*P*=0.0001, Spearman correlation coefficient=0.42). Also, based on result Table 2 there was a significant relationship between application of knowledge and knowledge management practices (*P*=0.0001, Spearman correlation coefficient=0.55). The results Jenatabadi et al. showed that had positive relationship between knowledge management and organizational performance (OP) [1]. Based on result Allameh and Abbas there is a strong, positive and significant relationship between knowledge management practices and innovation and knowledge of leadership [2]. Also, the obtained results are consistent with the results from previous studies by Zohoori et al. and Manafi et al. [3], [4]. Like many other similar studies in the field of management, the findings of this research face a serious challenge, which is the limitation of generalization. It means that while a few sub companies with unique circumstances were sampled for the study, the results can be generalized as different types of organizations.

**Table 1:** Demographic characteristics of employees in the Kurdistan food industry.

**Parameter**	**Characteristics**	**Number (In percent)**
Sex	Men	273 (86.4%)
	Female	43 (13.6%)
Age group	20–25	16 (5.1%)
	25–30	48 (15.2%)
	30–35	35 (11.1%)
	35–40	123 (38.9%)
	40–45	61 (19.3%)
	45–50	19 (6%)
	More than 50	14 (4.4%)
Educational qualification	Under the diploma	19(6%)
	Diploma	34(10.8%)
	Associate Degree	20(6.3%)
	Bachelor	229(72.5%)
	Master's degree and higher	14(4.4%)
Work experience	1–5	112 (35.4%)
	5–10	138 (43.7%)
	10–15	24 (7.6%)
	15–20	18 (5.7%)
	20–25	12 (3.8%)
	More than 25	12 (3.8%)

**Table 2:** The relationship between knowledge leadership and knowledge management practices in the Kurdistan food industry.

**Variable**	**The statistics**	**knowledge management practices**
**Knowledge driven leadership**	Spearman correlation coefficient	0.42
	P Value	0.0001
**Application of knowledge**	Spearman correlation coefficient	0.55
	P Value	0.0001

## 2 Experimental design, materials and methods

### 2.1 Study area description

Food industries of all areas (North, South, East, West, and Center) of the Kurdistan province (west of Iran) were selected. Iran is one of the most country in Middle East (ME) [5,^6^,7]. The Kurdistan province, with a population of 1,600,000 people, is one of the most mountainous (hilly) regions of Iran and has a generally mild and quite pleasant climate throughout the spring and summer. Also, this region has winters with heavy snowfalls. The province of Kurdistan is 28,817 km^2^ in area. Winters have a long period of time and can be very cold with heavy snowfalls.

### 2.2 Experimental design, materials and methods

22 food industries were chosen from Kurdistan province, Iran. 316 employees in the Kurdistan FIs participated in this study. In this study, data were gathered from all Food industries (Including Dairy, cold storage, flour, poultry, gum, pasta, mineral water, ice and soft drinks) as well as a researcher-made questionnaire (based on the Donate and Sanchez de Pablo, 2014) including the demographic data (e.g. age, sex, educational qualification and experience) and questions which were related to the causes of the improvement of the knowledge of leadership had been chosen [8,^9^,10]. There was a meaningful relationship between knowledge-based leadership and KM activities in the food industry of Kurdistan province. Then, the collected data were coded and entered into SPSS version 16. Data analysis was performed, using SPSS-16. Data were analyzed, applying descriptive and statistical tests including independent *t*-test and chi-square.
