# Supplementary material for: The relationship between knowledge of leadership and knowledge management practices in the food industry in Kurdistan province, Iran
Source: Data Brief. 2017 Sep 22;15:155–9. doi: 10.1016/j.dib.2017.09.031 (PMC5678737; doi:10.1016/j.dib.2017.09.031)
Supplement: Supplementary file 1 — Supplementary material [file mmc1.doc]

**The relationship between knowledge of leadership and knowledge management practices in the food industry in Kurdistan province, Iran**

**Conflicts of Interest**

Authors have no conflicts of interest.

**Acknowledgment**

This work was part of a funded MS thesis of Hafez Mohammadi, a student at Sanandaj Branch, Islamic Azad University. The authors would like to thank Sanandaj Branch, Islamic Azad University for providing financial support for this research.

**Funding/Support**

This work was part of a funded MS thesis of Hafez Mohammadi, a student at Sanandaj Branch, Islamic Azad University. The authors would like to thank Sanandaj Branch, Islamic Azad University for providing financial support for this research.
